# Psychological and work-related outcomes after inpatient multidisciplinary rehabilitation of chronic low back pain: a prospective randomized controlled trial

**DOI:** 10.1186/s40359-019-0282-3

**Published:** 2019-02-15

**Authors:** P. Hampel, A. Köpnick, S. Roch

**Affiliations:** Institute of Health, Nutrition, and Sport Sciences, Europa-Universität Flensburg, Auf dem Campus 1, 24943 Flensburg, Germany

**Keywords:** Chronic low back pain, Depressive symptoms, Work ability, Inpatient multidisciplinary rehabilitation, Cognitive-behavioral treatment

## Abstract

**Background:**

This study investigated the long-term effects (12 months post-rehabilitation) of a standard inpatient multidisciplinary rehabilitation program for patients with chronic low back pain (CLBP), in which a control group (CG) received pain competence training and an intervention group (IG) received combined pain competence and depression prevention training.

**Methods:**

In this prospective control group study with cluster-block randomization, a total of *n* = 583 patients were included into per protocol analyses. To examine the effects of rehabilitation on depressive symptoms, pain self-efficacy, and work ability, patients were stratified in repeated-measures analyses of variance by treatment condition (IG vs. CG), level of depressive symptoms (low vs. high), and time of assessment (pre, post, 6, and 12 months after rehabilitation). The impact of each treatment on pain-related days of sick leave (DSL; dichotomized into ≤ vs. > 2 weeks) was determined separately by conducting non-parametric analyses. Multiple imputations (*n* = 1225) confirmed the results. Effects were interpreted if clinical significance was given.

**Results:**

Only patients with high levels of depressive symptoms showed long-term improvements in depressive symptoms and self-efficacy. Long-term improvements in work ability index and mental work ability item were restricted to the IG. Furthermore, long-term effects on pain-related DSL were ascertained by per protocol and multiple imputation analyses only for the IG.

**Conclusions:**

Patients with high levels of depressive symptoms showed improvements in depressive symptoms and self-efficacy, supporting the psychological effectiveness of both interventions. However, the beneficial long-term effects of rehabilitation on work ability and pain-related DSL among the IG support implementation of combined pain competence and depression prevention training.

**Trial registration:**

DRKS00015465 (German Clinical Trial Register DRKS); date of registration: 03.09.2018.

**Electronic supplementary material:**

The online version of this article (10.1186/s40359-019-0282-3) contains supplementary material, which is available to authorized users.

## Introduction

Chronic low back pain (CLBP) is among the most common diseases in Western countries [1] and induces high medical as well as psychological and social costs [2]. There is a common understanding that a biopsychosocial perspective has to be applied to explain the etiology and treatment of CLBP appropriately [3]. Prior research provided strong evidence for the effectiveness of multimodal and multidisciplinary treatment of CLBP, in which psychological treatment elements were incorporated [4]. Moreover, those multimodal and multidisciplinary approaches were more effective than standard medical treatment, usual care, or physical treatment alone [5].

However, based on the major impact of psychological processes in pain chronification [3, 6–9], psychological treatment elements were more focused on the modification of pain-related fear-avoidance beliefs and maladaptive pain coping and did not show sustainable beneficial effects on mental symptoms. For instance, in a German study, patients with CLBP improved from comprehensive pain management training 12 months after inpatient multidisciplinary rehabilitation in pain coping, but not in pain self-efficacy nor in depressive symptoms [10]. Previous multimodal and multidisciplinary approaches neglected the aggravation of pain chronification caused by co-existing mental disorders such as major depression [11]. Thereby, strong evidence has been provided for the manifestation of depressive symptoms in consequence of chronic pain [12]. However, a reciprocal relationship has been supported by more current results [13]. Hence, protective factors for the development of depressive symptoms should also be addressed to prevent further pain chronification on the one hand and the development of mental disorders on the other hand.

Based on this empirical evidence, Hampel et al. [14–16] developed cognitive-behavioral management training for pain competence and depressive symptoms for patients with CLBP and subclinical and clinical depressive symptoms but who did not fulfill criteria for depression according to ICD-10. The module of pain competence training consisted of four 60-min group sessions guided by a psychotherapist and was designed in accordance with evidence-based models of fear-avoidance, self-efficacy, and stress-diathesis (for a review of psychological models, see [3]). Thus, psychological elements sought to treat pain-related fear-avoidance beliefs and improve stress and pain management in order to promote patients’ self-management competencies and self-efficacy expectations.

In contrast, the module of depression prevention training comprised five guided 60-min group sessions and was based on Beck’s cognitive theory of depression ([17], for a current review, see [18]). Hence, enhancement of the activity level, cognitive restructuring, and social skills training were incorporated. Additionally, pain-related cognitions, emotions, and behaviors, which were only briefly discussed in the pain competence training, were elaborated more deeply and functional behavior was practiced. Finally, maladaptive coping was explored and adaptive coping strategies were practiced. Thereby, reflection on stressors and application of adaptive situation-adapted coping strategies were focused on family and work-related conflicts, which have been shown to have a recent impact in onset and maintenance of LBP [19].

Both psychological modules were implemented into standard inpatient rehabilitation at two clinics, which were focused on traditional orthopedic rehabilitation. Due to the clientele of the pension insurance company, the sample consisted mainly of patients with lower education (68%; 22% middle, 4% high, 6% missing data). The intervention group (IG) was treated with both modules and compared to a control group, to whom the pain competence training was applied only. In sum, the IG showed significant sustainable psychological effects in orthopedic rehabilitation compared to pain competence training without prevention of depressive symptoms.

In the present study, this training was optimized with regard to didactic methods and manualized for evaluation [20]. The study was carried out in four clinics, which were focused on behavioral-medical rehabilitation for patients with CLBP and higher stress levels. Typically, in the setting of behavioral-medical rehabilitation in comparison with traditional orthopedic rehabilitation, a more multi-professional approach is applied and explicit psychological treatment elements are delivered (cf. [10]). To ensure the evaluation of effectiveness, treatments were implemented into routine rehabilitation. Thereby, the clientele of one clinic was comparable with the former sample [14–16]. The remaining three clinics treated patients with higher levels of education. While the amount of the pain competence training was not modified, the depression prevention training was reduced to four sessions in order to facilitate the implementation into the restricted time schedule. In order to have sufficient time for more interactive treatment elements, all sessions were expanded to 75 min. Because patients in the previous study were not well motivated to perform the homework assignments, unguided group workshops after each session were set up, in which the assigned exercises had to be completed. In addition, those group workshops were aimed to enhance the patients´ self-empowerment by encouraging them to practice self-management techniques on their own. Finally, current evidence for the beneficial treatment effects of acceptance and commitment models among patients with chronic pain [3] suggested the implementation of mindfulness-based training elements such as sensory perception and relaxation (Additional file 1: Table S1).

Previous quantitative analyses revealed similar short- and mid-term effects on depressive symptoms, anxiety, and pain from the combined pain competence and depression prevention training compared to treatment-as-usual with pain competence training only [21]. Nevertheless, analyses of qualitative interviews showed that the combined training was more appreciated by patients [22]. Moreover, patients who received combined training reported higher self-efficacy and a better biopsychosocial perspective.

The aim of this multi-center study was to analyze long-term effects of the modified combined cognitive-behavioral pain competence and depression prevention training in a different rehabilitation setting with different sample characteristics compared to the prior bi-center study. For this purpose, its effectiveness was investigated on depressive symptoms as a primary outcome. Moreover, self-efficacy was assessed as a secondary outcome, which is a core outcome of clinical trials [23], can be changed by cognitive-behavioral treatment [24], and is a strong predictor of functional chronic pain outcome and recovery [25]. Furthermore, work ability, which has been found to be a strong predictor of participation in working life [26], and days of sick leave (DSL) were measured as work-related (secondary) parameters [23]. It was expected that the newly developed program would elicit increased and stable improvements in the rehabilitation outcomes.

## Methods

### Design and procedure

A randomized controlled trial with cluster-block randomization was conducted. In the context of block randomization, the intervention condition was always carried out in two clinics and the control condition was simultaneously carried out in the other two clinics in order to control for seasonal effects. In addition, conditions were alternated every two months in terms of cluster randomization and an equal number of the two conditions were performed in each clinic [27]. A 2x2x4 repeated-measures design was realized with *treatment condition* and *level of depressive symptoms* as between-subjects factors and *time of assessment* as a within-subjects factor. The *treatment condition* consisted of the control group (CG; *n* = 288), who received pain competence training, and the intervention group (IG; *n* = 295), who received combined pain competence and depression prevention training. The *level of depressive symptoms* was assessed with the German version of the Center for Epidemiological Studies Depression Scale (CES-D; [28]), with a cut-off value of > 22 to separate the persons with low and high levels of depressive symptoms. To control for response bias, the sum of 16 negative mood items was subtracted by the sum of 4 positive (to be reversed) mood items multiplied by 4. Following Hautzinger et al. [28], questionnaires with a difference of ≤28 have to be excluded. The within-subjects factor *time* consisted of four sample points: pre (t0), post (t1), 6 months (t2), and 12 months (t3) after rehabilitation.

On the basis of previous studies and our own preliminary study [14–16], which revealed an intergroup effect in the per protocol (PP) analyses of *d* = .38 for the 24-month follow-up, a small effect size in the primary outcome measure “depressive symptoms” was assumed for the 24-month follow-up test between the IG and CG for the respective subgroups of depression. For this effect size, with an alpha level of .05, two-sided testing, and a desired power level of 1-β = 0.8, a prior power analysis using G*Power yielded a sample size of *n* = 176 for each of the four study groups. In the previous 24-month follow up-study, the dropout rate was 37%. Conservatively, a dropout rate of 40% and a response rate of 70% were calculated, so that a total sample of approximately *n* = 1173 patients enrolled at the time of measurement prior to rehabilitation was sought. For the present analysis, only data from the 12-month follow-up were analyzed, as in the 24-month follow-up correcting for missing data in the work ability score by multiple imputations (MI) failed due to extreme amount of missing data.

Patients were informed about the study during the first physical consultation in the clinic and were allocated to the treatment condition according to the week of arrival in the clinic. Allocation of the four clinics to the sequence of treatment condition took place according to a randomized Latin square design to ensure a balanced design. The assignment was conducted by an independent doctoral student at the Europa-Universität of Flensburg. Thus, the physicians and nursing staff at the clinics were blinded to the patients’ group assignments. It was not applicable to blind the therapists and patients, as the amount and contents of the treatment revealed the allocation to the groups. Recruitment took place from October 2014 until December 2015 and was finished when the expected sample size was achieved.

All data (except for the grade of chronicity, which was assessed by the physician during the first consultation) were collected using questionnaires filled in by the participants. Informed consent was obtained from all participants included in the study. This study had received full approval of the ethical review board of the German Psychological Society (DGPs) and was conducted in accordance with the 1964 Helsinki Declaration and its later amendments.

### Participants

A total of *n* = 583 participants were recruited in four inpatient rehabilitation clinics in Germany and included in per protocol analyses. The age ranged from 28 to 64 with a mean age of 53.3 years (*SD* = 6.1), 81.8% were female, and the mean pain duration was 15.2 years (*SD* = 10.8; Table 1). Moreover, 85.42% of the patients in the CG and 87.12% of the patients in the IG were employed at the beginning of the rehabilitation. Between-subjects Chi^2^- and *t*-tests did not show significant difference between the treatment conditions.

**Table 1:** Participants’ baseline characteristics and subjective rehabilitation success for both treatment conditions

Variable	Control group (*n* = 288)	Intervention group (*n* = 295)
Socio-demographic data		
Age [in years] (mean ± *SD*)	53.25 ± 6.09	53.26 ± 6.03
Gender, females no. (%)	235 (81.60%)	242 (82.03%)
Married no. (%)	180 (62.50%)	190 (64.41%)
Educational level (%)		
− low	58 (20.71%)	58 (19.93%)
− middle	143 (51.07%)	136 (46.74%)
− high	79 (28.21%)	97 (33.33%)
Work-related data		
Employed no. (%)	246 (85.42%)	257 (87.12%)
Days of sick leave due to pain in the last 3 months, more than 2 weeks no. (%)	133 (46.18%)	148 (50.17%)
Work ability score (WAI) (mean ± *SD*)	27.05 ± 8.47	26.41 ± 8.26
Pain history		
Pain duration [in years] (mean ± *SD*)	14.64 ± 9.98	15.73 ± 11.59
Pain locations no. (mean ± *SD*)	5.15 ± 2.53	5.35 ± 2.44
Average pain intensity (mean ± *SD*)	4.90 ± 1.90	4.91 ± 1.79
Grade of chronicity (MPSS) no. (%)		
I	69 (24.82%)	71 (25.00%)
II	133 (47.84%)	152 (53.52%)
III	76 (27.34%)	61 (21.48%)
Psychological status		
Depressive symptoms (CES-D) (mean ± *SD*)	23.82 ± 11.43	22.63 ± 10.84
Pain self-efficacy (PSEQ) (mean ± *SD*)	38.32 ± 11.29	38.99 ± 12.04
Subjective rehabilitation success (mean ± *SD*)	4.14 ± 1.32	4.27 ± 1.25

Chi^2^- and *t-*tests revealed no differences between control and intervention group

*WAI* work ability index, *MPSS* Mainz Pain Staging System, *CES-D* Center for Epidemiological Studies Depression Scale, *PSEQ* pain self-efficacy questionnaire, range of depressive symptoms: 0–60, range of pain self-efficacy: 10–60, work ability score: impaired (7–27) to excellent (44–49), pain intensity: ‘*no pain*’ (0) to ‘*pain as bad as could be*’ (10), subjective rehabilitation success: *‘very good’* (1) to ‘*insufficient’* (6)

The inclusion criteria were an age between 20 and 65 years prior to rehabilitation, a diagnosis of CLBP lasting at least 6 months (ICD-10: M51, M53, M54), informed consent for participation, and German language skills. Patients were excluded if they underwent surgery or had had an accident in the last 6 months before rehabilitation, had somatic diseases inducing back pain, were pregnant, had infections, had cardiovascular or metabolic diseases affecting rehabilitation, or had a serious psychiatric disorder.

### Treatment

The evaluation of the combined pain competence and depression prevention training was embedded in a standard inpatient multidisciplinary rehabilitation in four German clinics lasting three to four weeks. Four modules of pain competence training were delivered to both treatments, but the IG also received four modules of depression prevention training. All eight modules consisted of 75-min group interventions guided by a psychotherapist and a 25-min group workshop without a psychotherapist.

### Outcome measures

For the present analysis, the primary outcome and five secondary outcomes were selected. Depressive symptoms were measured by the CES-D, showing a good internal consistency in the present sample (α = .91), similar to the normative samples of Hautzinger et al. (α = .82, .92; [28]). The confidence of the patients in their ability to perform several activities despite pain was assessed by the 10-item German version of the pain self-efficacy questionnaire (PSEQ; [24, 29]). In contrast to the original version, pain self-efficacy must be evaluated on a six-point scale (1 = “*not at all confident*” to 6 = “*completely confident*”). A sum score was calculated with higher scores indicating higher self-efficacy (range 10–60), showing a good internal consistency (α = .94), similar to Mangels et al. ([29]; α = .93).

The work ability index (WAI; [30] measures the work ability taking into account physical and mental parts of work as well as different diseases and their impact on work ability. The WAI has a range from 7 to 49 with higher scores indicating higher work ability. In addition to the WAI, two single items asking for physical and mental job requirements were analyzed in order to detect differential rehabilitation effects on these two domains. The German short version of the WAI [31] was not filled in immediately after rehabilitation to avoid redundancy with the pre-assessment. Good psychometric quality has been shown for the German version [26].

*Pain-related DSL* in the last three months were dichotomized as up to or more than 2 weeks [14, 32]. This outcome measure was only reported by participants who were employed at the pre-assessment and at 12-month follow-up.

### Statistical analyses

For the CES-D and PSEQ, univariate two-way repeated-measures analyses of variance (ANOVA) was conducted with *treatment condition* (CG, IG) and *level of depressive symptoms* (low, high) as between-subjects factors and *time of assessment* (t0, t1, t2, t3) as a within-subjects factor. For the WAI (total score), only three sample points could be included into the univariate two-way repeated-measures ANOVA, as no post-assessment took place (t0, t2, t3). Moreover, a multivariate repeated-measures ANOVA was performed for the two single WAI items assessing the physical and mental work ability. Additionally, pairwise comparisons corrected by Bonferroni were performed to detect mean differences.

Furthermore, non-parametric analyses were carried out; DSL were analyzed with Friedman’s ANOVA separately for the CG and IG, followed by Wilcoxon signed-rank tests.

Analyses were conducted with SPSS Version 24 (SPSS Inc., Chicago, USA). The two-tailed significance level was set at *p* < .05 for all calculations due to the explorative character of the analyses. The effect sizes of the ANOVAs were interpreted as small (η^2^ = .01), medium (η^2^ = .06), or large (η^2^ = .14; [33]). For the between- and within-group effects, effect sizes using Cohen’s *d* were calculated and interpreted as small (*d* = .20), medium (*d* = .50), or large (*d* = .80). Effect sizes for Pearson product-moment correlations (*r*) were interpreted as small (*r* = .10), medium (*r* = .30), and large (*r* = .50; [33]).

PP results were validated by calculations after MI (*n* = 1225). The 10 imputations substituted single missing values as well as missing data due to dropout from the study. Due to a significant result on Little’s Missing Completely at Random (MCAR) test and as the power of testing increased with multiple imputations, these analyses were considered only as validation of the PP results. In addition, only results with at least a small effect size were interpreted (i.e., η^2^ > .01, *d* > .20, *r* > .10).

## Results

### Dropout analyses

**Fig. 1:**
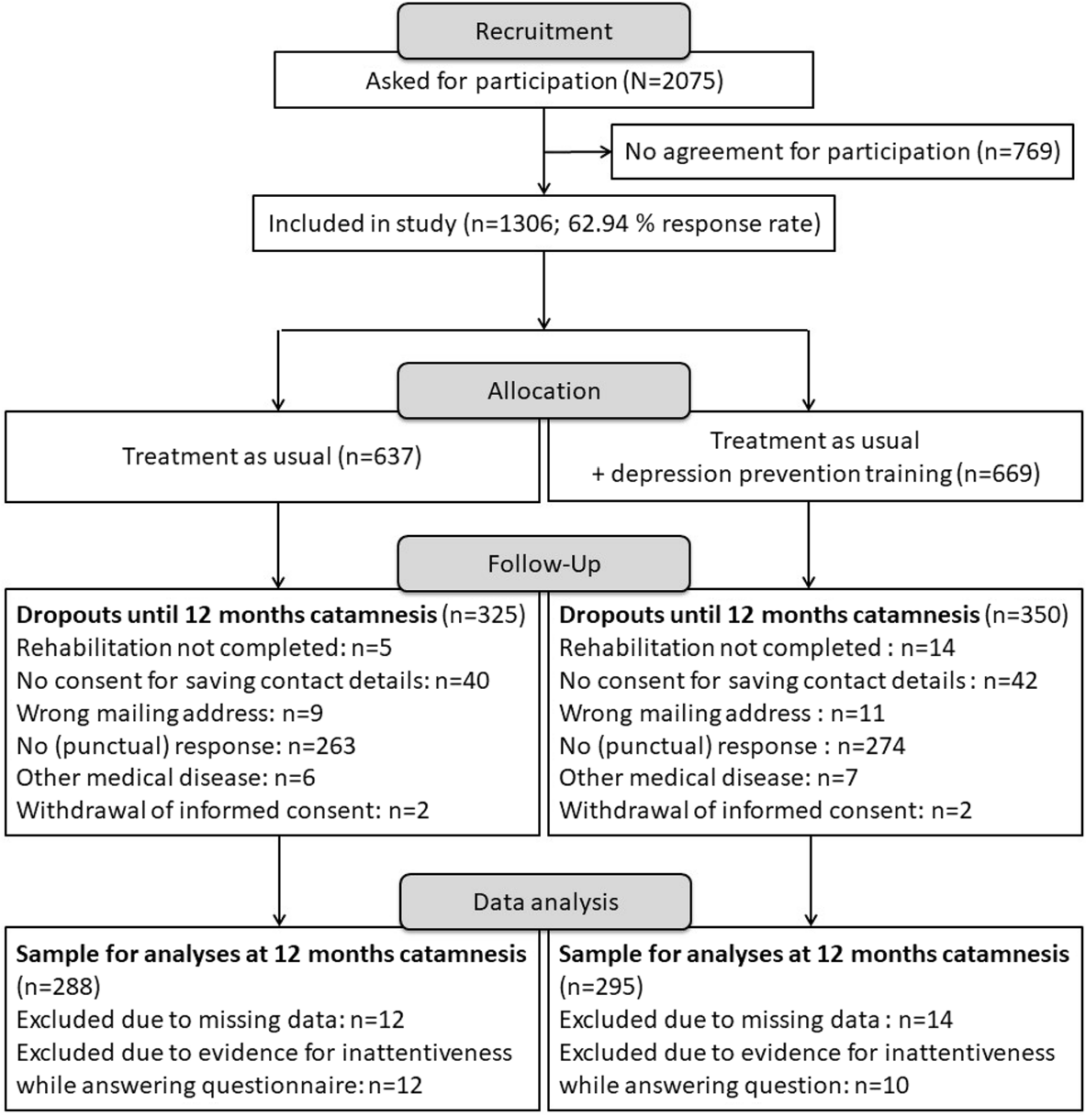
Flowchart of sample sizes for control and intervention group

A total of *n* = 2075 patients were asked to take part in the study; 769 patients did not agree to participate. Figure 1 depicts that the calculated total sample size of *n* = 1173 was marginally exceeded by the observed total sample size of *n* = 1306. In total, 675 dropped out up to the 12-month follow-up. Due to incomplete data for the CES-D score or because of evidence of response bias, 48 participants were excluded from the analyses. Thus, data from *n* = 583 participants were analyzed via the PP method.

Dropout rates did not depend on treatment condition (χ^2^(1) = 0.22, *p* = .639). However, the patients who dropped out were more often male, not married, and reporting more than 14 DSL and were less often employed. Additionally, they were younger, reported a shorter pain duration, had lower scores in the PSEQ and WAI, and reported a higher average pain intensity.

### Rehabilitation outcome

The following reports of rehabilitation effects are focused on the main and interaction effects of time.

#### Effects on psychological measures and work ability

##### Level of depressive symptoms by time

Univariate ANOVA yielded a simple interaction for depressive symptoms as well as pain self-efficacy (Table 2). In the long-term, only participants with high levels of depressive symptoms had statistically and clinically significant benefits from rehabilitation (depressive symptoms: t0-t3_high level_: *p* < .001, *d* = − 1.26; pain self-efficacy: t0-t3_high level_: *p* < .001, *d* = 0.44; Additional file 1: Table S2).

**Table 2:** Repeated-measures ANOVA results for main and interaction effects of treatment condition (TC), level of depressive symptoms (DS), and time of assessment (T) for depressive symptoms, pain self-efficacy and subjective work ability (analyses per protocol)

Variable		Factors						
		TC	DS	TC x DS	T	TC x T	DS x T	TC x DS x T
	df _1,2_	1, 579	1, 579	1, 579	2.6, 1505.7	2.6, 1505.7	2.6, 1505.7	2.6, 1505.7
Depressive symptoms	F	0.72	370.81	0.15	229.92	0.77	58.84	1.37
	p	.397	**<.001**	.697	**<.001**	.495	**<.001**	.253
	η^2^	.001	.390	.000	.284	.001	.092	.002
Pain self-efficacy	*df*_1,2_	1, 559	1, 559	1, 559	2.8, 1552.0	2.8, 1552.0	2.8, 1552.0	2.8, 1552.0
	*F*	1.00	88.60	0.00	57.39	3.57	6.62	1.53
	*p*	.317	**<.001**	.987	**<.001**	**.016**	**<.001**	.207
	η^2^	.002	.137	.000	.093	.006	.012	.003
WAI score	*df*_1,2_	1, 507	1, 507	1, 507	1.8, 889.6	1.8, 889.6	1.8, 889.6	1.8, 889.6
	*F*	0.01	72.36	0.21	37.50	7.15	0.69	0.88
	*p*	.920	**<.001**	.647	**<.001**	**.001**	.483	.404
	η^2^	.000	.125	.000	.069	.014	.001	.002
WAI items (multivariate)	*df*_1,2_	2, 568	2, 568	2, 568	4, 2274	4, 2274	4, 2274	4, 2274
	*F*	0.57	56.08	2.26	35.94	4.64	1.44	1.13
	*p*	.566	**<.001**	.105	**<.001**	**.001**	.217	.339
	η^2^	.002	.165	.008	.059	.008	.003	.002
WAI item physical	*df*_1,2_	1, 569	1, 569	1, 569	1.9, 1096.6	1.9, 1096.6	1.9, 1096.6	1.9, 1096.6
	*F*	0.22	59.05	0.27	44.62	2.54	0.33	1.90
	*p*	.641	**<.001**	.605	**<.001**	.081	.709	.152
	η^2^	.000	.094	.000	.073	.004	.001	.003
WAI item mental	*df*_1,2_	1, 569	1, 569	1, 569	1.9, 1096.3	1.9, 1096.3	1.9, 1096.3	1.9, 1096.3
	*F*	0.32	105.68	2.15	56.54	9.07	1.78	0.24
	*p*	.574	**<.001**	.143	**<.001**	**<.001**	.171	.780
	η^2^	.001	.157	.004	.090	.016	.003	.000

*df*_1,2_ degrees of freedom, η^2^ eta-square (effect size), *WAI* work ability index

Bold effects *p* < .05

##### Treatment condition by time

Simple interactions were ascertained for the WAI (total score) and mental work ability (see Table 2). The IG showed an improvement in the WAI 12 months after rehabilitation (t0-t3: *p* < .001, *d* = 0.42; Additional file 1: Table S3). In contrast, the CG did not have a clinically significant improvement (*d* < .20). Regarding the mental work ability item, the IG improved significantly in the long-term with a medium effect size (*p* < .001; t0-t3: *d* = 0.55), while the CG did not show a clinically significant enhancement in mental work ability.

##### Main time effects

Due to higher interaction effects with time, only the main time effect on physical work ability can be interpreted (Table 2). Pairwise comparisons revealed a significant long-term improvement with a low effect size (*p* < .001; t0-t3: *d* = 0.28). All effects reported above were confirmed by MI analyses (Additional file 1: Table S4).

#### Effects of treatment condition on DSL

Friedman’s ANOVA revealed a significant change in pain-related DSL over time in the IG (χ^2^(2) = 45.79, *p* < .001). A subsequent Wilcoxon test showed a significant long-term effect (Table 3). MI analyses confirmed these effects (χ^2^(2) = 76.55, *p* < .001; Additional file 1: Table S5); fewer participants than expected reported a change in their pain-related DSL.

**Table 3:** Observed and expected frequencies of days of sick leave because of pain dichotomized in up to and more than 2 weeks at the beginning (t_0_) as well as 6 months (t_2_) and 12 months (t_3_) after rehabilitation for both treatment conditions (per protocol analyses; IG; *n* = 295 above; CG; *n* = 288)

IG			t_2_		
			≤ 2 weeks	> 2 weeks	% Total
t_0_	≤ 2 weeks	Observed (%)	119 (40.3%)	28 (9.5%)	49.8%
		Expected	95.7	51.3	
	> 2 weeks	Observed (%)	73 (24.7%)	75 (25.4%)	50.2%
		Expected	96.3	51.7	
		% Total	65.1%	34.9%	
IG			t_3_		
			≤ 2 weeks	> 2 weeks	% Total
t_0_	≤ 2 weeks	Observed (%)	124 (42.0%)	23 (7.8%)	49.8%
		Expected	106.1	40.9	
	> 2 weeks	Observed (%)	89 (30.2%)	59 (20.0%)	50.2%
		Expected	106.9	41.1	
		% Total	72.2%	27.8%	
CG			t_2_		
			≤ 2 weeks	> 2 weeks	% Total
t_0_	≤ 2 weeks	Observed (%)	116 (40.3%)	39 (13.5%)	53.8%
		Expected	92.6	62.4	
	> 2 weeks	Observed (%)	56 (19.4%)	77 (26.7%)	46.2%
		Expected	79.4	53.6	
		% Total	59.7%	40.3%	
CG			t_3_		
			≤ 2 weeks	> 2 weeks	% Total
t_0_	≤ 2 weeks	Observed (%)	113 (39.2%)	42 (14.6%)	53.8%
		Expected	93.1	61.9	
	> 2 weeks	Observed (%)	60 (20.8%)	73 (25.3%)	46.2%
		Expected	79.9	53.1	
		% Total	60.1%	39.9%	

In contrast, Friedman’s ANOVA revealed no significant changes in pain-related DSL in the CG (χ^2^(2) = 4.36, *p* = .113; Table 3). However, MI analyses showed significant changes in Friedman’s ANOVA results (χ^2^(2) = 22.92, *p* = .002) as well as a significant long-term effect on the Wilcoxon test (Additional file 1: Table S5). Summarizing the effects for the CG, fewer participants than expected reported a change in their DSL.

Descriptive analyses of the PP results indicated that especially the distributions 12 months after rehabilitation differed by treatment condition: More participants in the IG than in the CG improved (30.2% vs. 20.8%), and fewer worsened (7.8% vs. 14.6%) from pre-rehabilitation until 12 months post-rehabilitation. Overall, MI analyses revealed significant effects for both conditions, but effect sizes in the IG were larger than in the CG and PP analyses were not significant for the CG. Further descriptive analyses with post hoc chi^2^-test support that despite of a similar rate of employment at the beginning of the rehabilitation (see Table 1), the distribution of rate of employment differed significantly at the 12-month follow-up assessment to the disadvantage of the CG (IG vs. CG: employed: 85.4% vs. 77.8%; χ^2^(1) = 5.69, *p* = .017).

## Discussion

This paper predominantly sought to evaluate the long-term effects of a combined cognitive-behavioral pain competence and depression prevention training on depressive symptoms, pain self-efficacy, work ability, and pain-related DSL. Patients with high levels of depressive symptoms improved more from the rehabilitation in depressive symptoms and pain self-efficacy, pointing to the significant influence of depressive symptoms on CLBP [11]. Since pain self-efficacy is a mediator for the development of disability among patients with CLBP [34], pain self-efficacy should be promoted during rehabilitation.

However, the IG had more favorable results compared to the CG in the long-term in general work ability (WAI), mental work ability, and pain-related DSL. The results for work ability are particularly of interest because the IG seems to have demonstrated a differential effect on mental work ability but not on physical work ability. These selective effects can be attributed to the content of the additional depression prevention training in the IG ([20]; see Additional file 1: Table S1), which contained information, practice, and discussion as means of reducing emotional and work-related stress. These contents (e.g., activity management, cognitive restructuring or social competence) are common in cognitive-behavioral treatment of major depression that proved to be effective for treatment of depression [35], though in this study no differential treatment effects on depressive symptoms have been found. However, they may also directly increase work ability by changing the view on daily hassles and communication with colleagues. Here, especially the content about maladaptive coping of family and work-related conflicts may be important, which was only addressed in the IG. Likewise, the impact on general work ability seen in the IG may also have been evoked by changes in mental work ability, as mental work ability is a part of the overall work ability score. These hypotheses about differential impacts need to be further analyzed.

Moreover, similar to the earlier 1-year longitudinal study [14], only the IG experienced an impact on pain-related DSL, whereas there were no significant changes in the CG. This difference between treatment conditions increased across both follow up-assessments. Taking the results of MI analyses into account, the CG, too, showed significant effects, but with smaller effect sizes than the IG, which may be explained by the larger sample size in the MI analyses leading to more test power.

### Limitations

Dropout analyses revealed differences between dropped out patients and patients who stayed in the study. Most of these differences were taken into account during MI leading to the conclusion that dropout presumably did not bias effects that were confirmed by MI analyses. Moreover, effects concerning differences in CG and IG are unlikely to be biased by dropout because dropout was independent from treatment condition. Overall, conducted MI analyses did confirm most PP analyses, but some effects differed between both analyses. Nevertheless, interpretations were based on PP analyses with at least small effect sizes because of the significant Little’s MCAR test and increased power of testing in the MI analyses that might lead to significant effects even for small effect sizes.

The two treatment conditions differed in the amount of psychological treatment sessions provided. In the IG eight sessions were applied, instead of four sessions in the CG. The rehabilitation clinics were asked to attenuate the difference by offering more unspecific treatment elements such as relaxation to the CG. Descriptive analyses suggested that the difference of four sessions was not fully compensated. Thus, as Waterschoot et al. [36] concluded earlier in their systematic review, independent effects of dose variables has to be distinguished from content in future studies. Nevertheless, differential effects of conditions on the psychological and work-related outcomes lend support to the assumption that they might be explained by the content.

Further analyses drawing a subsample of patients with lower education confirmed former beneficial results of the combined treatment on depressive symptoms [14, 16, 37]. Several reviews highlighted the relevance of social factors and determinants for the development of LBP and its chronification [19, 38]. Thus, the present findings are limited to patients with higher levels of education and further investigation of differential effects of social factors on rehabilitation outcome of the combined treatment (Debora) is recommended.

## Conclusions

This study supported the hypothesis that combined cognitive-behavioral management training for pain competence and depressive symptoms (Debora) is more effective in increasing (mental) work ability and reducing pain-related DSL than a treatment-as-usual including pain competence training only. The preliminary results of the differential effects of level of education on psychological outcomes need to be replicated. Overall, the effectiveness of multidisciplinary treatments for CLBP applied by a multi-professional team was confirmed by the sustainable effects of both interventions on physical work ability among all patients and on depressive symptoms and pain self-efficacy among patients with high levels of depressive symptoms.

## Additional file

Additional file 1: **Table S1.** Modules’ description of the pain competence and depression prevention training. **Table S2.** Means (*M*), standard deviations (*SD*), within-group effect sizes Cohen’s *d* (ES), and pairwise comparisons (*p*) for the interaction effect of level of depressive symptoms and time for depressive symptoms (CES-D) and the Pain Self-Efficacy Questionnaire (PSEQ). **Table S3.** Means (*M*), standard deviations (*SD*), within-group effect sizes Cohen’s *d* (ES), and pairwise comparisons (*p*) for the interaction effect of treatment condition and time for the Work Ability Index (WAI). **Table S4.** Repeated measures ANOVA results for main and interaction effects of treatment condition (TC), level of depressive symptoms (DS), and time of assessment (T) for pain self-efficacy and subjective work ability (analyses after multiple imputations). **Table S5.** Observed and expected frequencies of days of sick leave because of pain dichotomized in up to and more than 2 weeks at the beginning (t_0_) as well as 6 months (t_2_) and 12 months (t_3_) after rehabilitation for both treatment conditions (multiple imputation analyses; above: IG; *n*=627; below: CG; *n*=598). (DOCX 60 kb)

## Abbreviations

ANOVA: Analysis of variance
CES-D: Center for Epidemiological Studies Depression Scale
CG: Control group
CLBP: Chronic low back pain
DGPs: Deutsche Gesellschaft für Psychologie
DSL: Days of sick leave
ESM: Electronic supplementary material
ICD: International Statistical Classification of Diseases and Related Health Problems
IG: Intervention group
MCAR: Little’s Missing Completely at Random
MI: Multiple imputation
PP: Per protocol
PSEQ: Pain self-efficacy questionnaire
SPSS: Statistical Package for the Social Sciences
WAI: Work ability index
