# Supplementary material for: Psychological and work-related outcomes after inpatient multidisciplinary rehabilitation of chronic low back pain: a prospective randomized controlled trial
Source: BMC Psychol. 2019 Feb 15;7:6. doi: 10.1186/s40359-019-0282-3 (PMC6377771; doi:10.1186/s40359-019-0282-3)
Supplement: Supplementary file 1 — Table S1. Modules’ description of the pain competence and depression prevention training. Table S2. Means (M), standard deviations (SD), within-group effect sizes Cohen’s d (ES), and pairwise comparisons (p) for the interaction effect of level of depressive symptoms and time for depressive symptoms (CES-D) and the Pain Self-Efficacy Questionnaire (PSEQ). Table S3. Means (M), standard deviations (SD), within-group effect sizes Cohen’s d (ES), and pairwise comparisons (p) for the interaction effect of treatment condition and time for the Work Ability Index (WAI). Table S4. Repeated measures ANOVA results for main and interaction effects of treatment condition (TC), level of depressive symptoms (DS), and time of assessment (T) for pain self-efficacy and subjective work ability (analyses after multiple imputations). Table S5. Observed and expected frequencies of days of sick leave because of pain dichotomized in up to and more than 2 weeks at the beginning (t0) as well as 6 months (t2) and 12 months (t3) after rehabilitation for both treatment conditions (multiple imputation analyses; above: IG; n=627; below: CG; n=598). (DOCX 60 kb) [file 40359_2019_282_MOESM1_ESM.docx]

**Table ESM1** Modules’ description of the pain competence and depression prevention training

| Pain competence training modules | Depression prevention training modules |
| --- | --- |
| 1. *Behavioral patterns and pain*:  Presentation of the reciprocal relation of behavioral patterns and pain based on the biopsychosocial model, differentiation of favorable and unfavorable behavior. | 1. *Activity management*:  Implementing favorable behavior, presentation of the principle of balanced activities, monitoring by an activity-emotions-pain-protocol. |
| 2. *Emotions and pain*:  Presentation of the reciprocal relation of emotions and pain, differentiation of pain reducing and pain inducing emotions. | 2. *Pain communication*:  Discussion of the role of (non-verbal) communication, reflection of the patients’ own pain communication. |
| 3. *Cognitions and pain*:  Presentation of the reciprocal relation of cognitions and pain, differentiation of pain reducing and pain inducing cognitions, explanation of the pain-reducing effect of distraction. | 3. *Cognitive restructuring*:  Introduction to automatized thoughts, discussion of minimizing and catastrophizing pain, cognitive restructuring of pain-related irrational and negative beliefs by ABC-schema. |
| 4. *Stress and pain*:  Presentation of the reciprocal relation of stress and pain, illustration of the development of stress and of personal stress situations, stress reactions, and adaptive coping strategies. | 4. *Stress management*:  Differentiation of adaptive and maladaptive coping, practice of adaptive coping strategies, social skills training, and closure of the training together with the trainer (workshop takes place before module). |

**Table ESM2** Means (*M*), standard deviations (*SD*), within-group effect sizes Cohen’s *d* (ES), and pairwise comparisons (*p*) for the interaction effect of level of depressive symptoms and time for depressive symptoms (CES-D) and the Pain Self-Efficacy Questionnaire (PSEQ)

|  | | **Descriptive statistics** | | | | | | | |  | | | | | | | | | **Dependent comparisons** | | | | | | | |
| --- | --- | --- | --- | --- | --- | --- | --- | --- | --- | --- | --- | --- | --- | --- | --- | --- | --- | --- | --- | --- | --- | --- | --- | --- | --- | --- |
|  |  | **Low level of depressive symptoms** | | | | | **High level of depressive symptoms** | | | | |  | **Low level of depressive symptoms** | | | | | | | **High level of depressive symptoms** | | | | | | |
|  |  | **t_0_** | **t_1_** | **t_2_** | **t_3_** | **t_0_** | | **t_1_** | **t_2_** | **t_3_** |  | | | **t_0_-t_1_** | **t_0_-t_2_** | **t_0_-t_3_** | **t_1_-t_2_** | **t_1_-t_3_** | **t_2_-t_3_** | | **t_0_-t_1_** | **t_0_-t_2_** | **t_0_-t_3_** | **t_1_-t_2_** | **t_1_-t_3_** | **t_2_-t_3_** |
| **CES-D** | *M* | 14.00 | 8.39 | 13.28 | 14.05 | 32.25 | | 16.84 | 23.64 | 24.21 | ES | | | -.87 | -.11 | .01 | .56 | .65 | .07 | | -2.41 | -1.35 | -1.26 | .786 | .851 | .05 |
|  | *SD* | 6.41 | 8.67 | 10.85 | 11.33 | 6.40 | | 8.66 | 10.84 | 11.31 | *p* | | | <.001 | .228 | .93 | <.001 | <.001 | .133 | | <.001 | <.001 | <.001 | <.001 | <.001 | .262 |
| **PSEQ** | *M* | 43.76 | 46.68 | 46.68 | 45.75 | 33.72 | | 39.60 | 39.35 | 38.38 | ES | | | 0.28 | 0.27 | 0.19 | 0.00 | -0.08 | -0.08 | | 0.56 | 0.53 | 0.44 | -0.02 | -0.11 | -0.08 |
|  | *SD* | 10.61 | 11.16 | 11.87 | 12.24 | 10.58 | | 11.13 | 11.84 | 12.19 | *p* | | | <.001 | <.001 | .001 | .991 | .091 | .060 | | <.001 | <.001 | <.001 | .632 | .027 | .049 |

*t_0_* beginning of rehabilitation, *t_1_* end of rehabilitation, *t_2_* 6 months after rehabilitation, *t_3_* 12 months after rehabilitation

**Table ESM3** Means (*M*), standard deviations (*SD*), within-group effect sizes Cohen’s *d* (ES), and pairwise comparisons (*p*) for the interaction effect of treatment condition and time for the Work Ability Index (WAI)

|  | | **Descriptive statistics** | | | | | |  | **Dependent comparisons** | | | | | |
| --- | --- | --- | --- | --- | --- | --- | --- | --- | --- | --- | --- | --- | --- | --- |
|  |  | **Control group** | | | **Intervention group** | | |  | **Control group** | | | **Intervention group** | | |
|  |  | **t_0_** | **t_2_** | **t_3_** | **t_0_** | **t_2_** | **t_3_** |  | **t_0_-t_2_** | **t_0_-t_3_** | **t_2_-t_3_** | **t_0_-t_2_** | **t_0_-t_3_** | **t_2_-t_3_** |
| **WAI Score** | *M* | 27.34 | 28.55 | 28.57 | 26.28 | 28.78 | 29.62 | ES | 0.15 | 0.16 | 0.00 | 0.32 | 0.42 | 0.09 |
|  | *SD* | 7.87 | 8.84 | 9.14 | 7.88 | 8.86 | 9.16 | *p* | .003 | .008 | .950 | <.001 | <.001 | .009 |
| **WAI item mental** | *M* | 2.89 | 3.15 | 3.04 | 2.74 | 3.23 | 3.21 | ES | 0.31 | 0.18 | -0.10 | 0.58 | 0.55 | -0.02 |
|  | *SD* | 0.85 | 1.01 | 1.00 | .85 | 1.01 | 1.00 | *p* | <.001 | .007 | .030 | <.001 | <.001 | .666 |

*t_0_* beginning of rehabilitation, *t_2_* 6 months after rehabilitation, *t_3_* 12 months after rehabilitation

**Table ESM4** Repeated measures ANOVA results for main and interaction effects of treatment condition (TC), level of depressive symptoms (DS), and time of assessment (T) for pain self-efficacy and subjective work ability (analyses after multiple imputations)

| Variable | | Factors | | | | | | |
| --- | --- | --- | --- | --- | --- | --- | --- | --- |
|  |  | TC | DS | TC x DS | T | TC x T | DS x T | TC x DS x T |
|  | *df* _1,2_ | 1, 1221 | 1,  1221 | 1,  1221 | 2.8, 3359.5 | 2.8, 3359.5 | 2.8, 3359.5 | 2.8,  3359.5 |
| Depressive symptoms | *F* | 1.35 | 927.09 | 0.28 | 367.00 | 0.58 | 109.34 | 0.93 |
|  | *p* | .284 | **< .001** | .678 | **< .001** | .676 | **< .001** | .555 |
|  | η^2^ | .001 | .431 | .000 | .231 | .000 | .082 | .001 |
| Pain self-efficacy | *df* _1,2_ | 1,  1221 | 1,  1221 | 1,  1221 | 2.8,  3453.8 | 2.8,  3453.8 | 2.8,  3453.8 | 2.8,  3453.8 |
|  | *F* | 2.56 | 217.83 | 0.44 | 102.69 | 1.11 | 8.88 | 0.91 |
|  | *p* | .119 | **< .001** | .537 | **< .001** | .416 | **< .001** | .489 |
|  | η^2^ | .002 | .151 | .000 | .078 | .001 | .007 | .001 |
| WAI score | *df* _1,2_ | 1,  1221 | 1,  1221 | 1,  1221 | 1.8,  2198.3 | 1.8,  2198.3 | 1.8,  2198.3 | 1.8,  2198.3 |
|  | *F* | 0.12 | 189.91 | 0.12 | 84.88 | 4.16 | 5.59 | 0.86 |
|  | *p* | .758 | **< .001** | .766 | **< .001** | .078 | **.020** | .438 |
|  | η^2^ | .000 | .135 | .000 | .065 | .003 | .005 | .001 |
| WAI items (multivariate) | *df* _1,2_ | 2,  1220 | 2,  1220 | 2,  1220 | 4,  4882 | 4,  4882 | 4,  4882 | 4,  4882 |
|  | *F* | 0.16 | 134.10 | 1.98 | 69.86 | 2.64 | 7.60 | 1.73 |
|  | *p* | .862 | **< .001** | .187 | **< .001** | .062 | **< .001** | .282 |
|  | η^2^ | .000 | .180 | .003 | .054 | .002 | .006 | .001 |
| WAI item  physical | *df* _1,2_ | 1,  1221 | 1,  1221 | 1,  1221 | 2.0,  2396.3 | 2.0,  2396.3 | 2.0,  2396.3 | 2.0,  2396.3 |
|  | *F* | 0.07 | 106.17 | 0.11 | 89.7 | 1.60 | 0.44 | 2.41 |
|  | *p* | .814 | **< .001** | .766 | **< .001** | .288 | .705 | .166 |
|  | η^2^ | .000 | .080 | .000 | .068 | .001 | .000 | .002 |
| WAI item  mental | *df* _1,2_ | 1,  1221 | 1,  1221 | 1,  1221 | 1.9  2347.7 | 1.9  2347.7 | 1.9  2347.7 | 1.9  2347.7 |
|  | *F* | 0.19 | 255.18 | 2.89 | 97.74 | 4.75 | 12.96 | 0.46 |
|  | *p* | .713 | **< .001** | .106 | **< 001** | **.021** | **< .001** | .682 |
|  | η^2^ | .000 | .173 | .002 | .074 | .004 | .010 | .000 |

*df*_1,2_ degrees of freedom, η^2^ eta-square (effect size), *WAI* work ability index.

Bold effects *p* < .05.

**Table ESM5** Observed and expected frequencies of days of sick leave because of pain dichotomized in up to and more than 2 weeks at the beginning (t_0_) as well as 6 months (t_2_) and 12 months (t_3_) after rehabilitation for both treatment conditions (multiple imputation analyses; above: IG; *n*=627; below: CG; *n*=598)

| **IG** | | | **t_2_** | | |
| --- | --- | --- | --- | --- | --- |
|  |  |  | **≤ 2 weeks** | **> 2 weeks** | **% Total** |
| **t_0_** | **≤ 2 weeks** | **Observed (%)** | 222.5 (35.5%) | 68.5 (10.9%) | 46.4% |
|  |  | **Expected** | 172.8 | 118.2 |  |
|  | **> 2 weeks** | **Observed (%)** | 149.9 (23.9%) | 186.1 (29.7%) | 53.6% |
|  |  | **Expected** | 199.6 | 136.4 |  |
|  |  | **% Total** | 59.4% | 40.6% |  |
| **IG** | | | **t_3_** | | |
|  |  |  | **≤ 2 weeks** | **> 2 weeks** | **% Total** |
| **t_0_** | **≤ 2 weeks** | **Observed (%)** | 232.1 (37.0%) | 58.9 (9.4%) | 46.4% |
|  |  | **Expected** | 194.0 | 97.0 |  |
|  | **> 2 weeks** | **Observed (%)** | 185.9 (29.6%) | 150.1 (23.9%) | 53.6% |
|  |  | **Expected** | 224.0 | 112.0 |  |
|  |  | **% Total** | 66.7% | 33.3% |  |
| **CG** | | | **t_2_** | | |
|  |  |  | **≤ 2 weeks** | **> 2 weeks** | **% Total** |
| **t_0_** | **≤ 2 weeks** | **Observed (%)** | 212.6 (35.6%) | 81.4 (13.6%) | 49.2% |
|  |  | **Expected** | 164.3 | 129.7 |  |
|  | **> 2 weeks** | **Observed (%)** | 121.5 (20.3%) | 182.5 (30.5%) | 50.8% |
|  |  | **Expected** | 169.8 | 134.2 |  |
|  |  | **% Total** | 55.9% | 44.1% |  |
| **CG** | | | **t_3_** | | |
|  |  |  | **≤ 2 weeks** | **> 2 weeks** | **% Total** |
| **t_0_** | **≤ 2 weeks** | **Observed (%)** | 214.0 (35.8%) | 80.0 (13.4%) | 49.2% |
|  |  | **Expected** | 176.7 | 117.3 |  |
|  | **> 2 weeks** | **Observed (%)** | 145.5 (24.3%) | 158.5 (26.5%) | 50.8% |
|  |  | **Expected** | 182.8 | 121.2 |  |
|  |  | **% Total** | 60.1% | 39.9% |  |
